# Tumor-infiltrating B lymphocytes as an efficient source of highly specific immunoglobulins recognizing tumor cells

**DOI:** 10.1186/1472-6750-7-70

**Published:** 2007-10-18

**Authors:** Emiliano Pavoni, Giorgia Monteriù, Daniela Santapaola, Fiorella Petronzelli, Anna Maria Anastasi, Angela Pelliccia, Valeria D'Alessio, Rita De Santis, Olga Minenkova

**Affiliations:** 1 Kenton Srl, c/o Sigma-Tau SpA, via Pontina, km 30.400, 00040 Pomezia (RM), Italy; 2 Immunology Department, Sigma-Tau SpA, via Pontina, km 30.400, 00040 Pomezia (RM), Italy

## Abstract

**Background:**

There is much evidence that tumor cells elicit a humoral immune response in patients. In most cases, the presence of antibodies in peripheral blood is detected only in small proportion of patients with tumors overexpressing the corresponding antigen. In the present study, we analyzed the significance of local humoral response provided by tumor-infiltrating lymphocytes in breast cancer patients.

**Methods:**

The ability of a patient's immune system to produce specific antibodies inside tumor tissue, capable of recognizing tumor cells, was explored through analysis of the oligoclonality of antibodies derived from tumor-infiltrating lymphocytes and construction of a series of recombinant antibody libraries in scFv format, derived from breast tumor-infiltrating B lymphocytes. These libraries and one from peripheral blood lymphocytes of a single breast cancer patient were panned against three purified surface tumor antigens, such as CEA, MUC1 and ED-B domain, and against intact MCF7 breast carcinoma cells.

**Results:**

Application of novel display vector, pKM19, allowed isolation of a large panel of breast cancer-specific antibodies against known tumor antigens, as well as against breast carcinoma cells. Reactivity of novel scFvs was confirmed by ELISA, immunohistochemistry, fluorescence staining and flow cytometry. We demonstrated that seven of ten primary breast tumor specimens, obtained using discarded surgical material, could be exploited as an appropriate source for generation of phage display libraries, giving highly specific antitumor antibodies which recognize heterologous tumor cells.

**Conclusion:**

Local humoral immune response within tumor tissue in breast cancer patients frequently has an oligoclonal character. Efficient selection of specific antitumor antibodies from recombinant antibody libraries, derived from such oligoclonal tumor-infiltrated B lymphocytes, indicates the presence of natural immune response against tumor antigens in these patients. The described method is very promising for development of antitumor antibodies, potentially useful for diagnostic and therapeutic approaches.

## Background

The discovery of monoclonal antibody technology [1] stimulated rapid development of targeted therapies against cancer. The use of monoclonal antibodies as a drug delivery vehicles, or trigger for human immune response is already an accepted method for therapeutic treatment of patients in modern clinical oncology [2,3]. However, initially promising mouse monoclonal antibodies induced development of anti-mouse immune antibody response (HAMA) in patients under repeated monoclonal antibody administration, thus limiting their application [4]. Recombinant DNA technology provides a cheap, useful alternative to monoclonal antibody production, allowing generation of large human recombinant antibody libraries displayed on the surface of filamentous phage and selection of specific human antibodies against desirable targets, useful for therapy [5-8]. Moreover, phage display also enables affinity maturation of antibodies *in vitro *through construction of mutant antibody libraries, giving clones of greater affinity [9,10].

The possibility of finding high-affinity binders in a recombinant antibody library depends on its quality, which is dependent on several factors, such as library size, diversity and source of immunoglobulin genes. It is known that various lymphoid tissues from immunized or nonimmunized donors, such as peripheral blood lymphocytes [11,12], spleen and bone marrow [13] and even metastasized or drained lymph node tissue from individuals with tumors [14-18] may serve as a source of specific antibody repertoire. Although naïve antibody libraries are more diverse and lead to isolation of antibodies with broad specificities, it is reasonable to suggest that construction of a recombinant antibody library from the immunoglobulin repertoire of someone affected by tumor can provide antibody fragments of higher binding affinity against specific tumor antigens. Early evidence that tumor-infiltrating B lymphocyte (TIL-B)-derived antibodies may also recognize tumor cells was obtained in the following ways: by production of human hybridomas derived from TIL, able to secrete tumor-specific antibodies [19,20]; B cell expansion of TIL from human tumor biopsies [21]; B cell expansion of melanoma-derived TIL, and following cloning of the scFv antibody with specific melanoma reactivity from single B cell clone [22]; and subcutaneous transplantation of human lung cancer tissue in immunodeficient mice [23,24], all of which suggest a specific function of TIL-B in the tumor. Recently, a rare type of breast cancer, classified as medullary carcinoma (MCB, medullary carcinoma of breast), characterized by strong lymphoplasmacytic infiltrates correlated with improved prognosis and patient survival, and cervical carcinoma, were investigated to understand the nature of tumor-infiltrated B lymphocytes through analysis of TIL-derived Ig repertoire [25-28]. A study of the molecular structure of variable antibody regions gave evidence of antigen-driven humoral immune responses in medullary breast carcinomas, as well as in cervical tumors. The oligoclonal predominance found in antibody genes derived from TIL indicated possible clonal selection of the Ig molecules against specific neoantigens overexpressing, or specifically expressing, in tumor tissue. Despite the very strong above-mentioned indications that tumor tissue is infiltrated with activated B cells, which may serve as a source of tumor-specific antibodies, in panning experiments performed against purified known tumor antigens, living tumor cells or frozen tissue sections, several research groups failed to select either a specific antibody discriminating between tumor and normal cells, or one reactive with cell-surface tumor antigens [26,27,29]. Only later, two different groups managed to identify specific antibodies recognizing tumor cells from this type of phage-display libraries [30,31].

Despite the limited success obtained by other research groups exploring the recombinant antibody libraries derived from TIL-B, we generated a series of TIL-B-based scFv libraries from breast tumors. We panned them, along with a peripheral blood lymphocyte (PBL)-derived library from a single breast tumor patient, on living MCF7 breast carcinoma cells, as well as on three purified tumor surface antigens, i.e., CEA (carcinoembryonic antigen) [32], MUC1 (epithelial mucin) [33] and ED-B domain of fibronectin [34]. A novel pKM19 vector [35], designed to provide relatively low expression levels of recombinant antibodies, thus reducing biological bias for their expression in *E. coli*, was used for generation of antibody libraries in scFv format. Cell-based panning and selection of scFv antibodies against purified antigens provided, in each case, a panel of highly specific antibodies from TIL- or PBL-derived scFv libraries.

## Methods

### Tissue and blood samples

Specimens of breast carcinoma and fresh peripheral blood from breast cancer patient EC23 were obtained from M. G. Vannini Hospital, Rome. All human biological samples were obtained through informed consent.

### Cell lines

The breast carcinoma cell lines MCF7 (ATCC nr: HTB-22), MDA-MB-468 (ATCC nr: HTB-132) were maintained in DMEM/F12, supplemented with 5% FBS and used for cell-based panning or for cell-ELISA. Immortal breast epithelial cells MCF10-2A (ATCC nr: CRL-10781) [36] were propagated according to manufacturer's instructions and used as negative controls in ELISA tests. Human foreskin fibroblasts (HFF) were cultivated in DMEM supplemented with 10% FBS and 1% L-glutamine.

### Purified tumor antigen proteins

Human CEA protein, purified from human colon carcinoma and liver metastases, was purchased from USBiological (#C1300-16, United States Biological, Swampscott, MA).

Biotinylated recombinant ED-B domain of fibronectin was obtained from Sigma-Tau, Pomezia, Rome.

Recombinant MUC1 protein was obtained in several steps. Two overlapping oligonucleotides KM358 5'-ACT TCA GCT CCG GAC ACC CGT CCG GCT CCG GGT TCC ACC GCT CCG CCG GCT CAC GGT GTC-3' and KM359 5'-CGG AGC CGG ACG GGT GTC CGG AGC TGA AGT GAC ACC GTG AGC CGG CGG AGC GGT GGA ACC-3', encoded for MUC1 20 amino acid repeat, were assembled in PCR-like process, in which 25 cycles of PCR amplification were performed with KM358 and KM359 primers in a concentration of 0.2 mM. The high-weight DNA band was then cut from agarose gel and ligated with a short adapter, obtained by annealing an oligonucleotide KM328 5'-CT AGT TCG TCG GGT TCG TCG GGA-3' with a phosphorylated KM329 5'-TCC CGA CGA ACC CGA CGA A-3', thus facilitating cloning of the blunt-end DNA fragment in the *Spe*I site. The resulting DNA fragment was purified from adapter excess, phosphorylated and cloned into digested and dephosphorylated pGEX-SN [37], derived from the pGEX-3× plasmid [38]. GST-fused MUC1 recombinant protein, containing a 107 amino acid MUC1 sequence, containing 5.3 repeats, was purified by using Glutathione Sepharose 4B (Amersham Biosciences, Uppsala, Sweden) according to manufacturer's instructions.

### Purification of peripheral blood lymphocytes

The lymphocytes were isolated from 10 mL of fresh peripheral blood mixed with anticoagulant by using Ficoll-Paque Plus (Amersham Pharmacia Biotech, Uppsala, Sweden) according to manufacturer's instructions. The mRNA was isolated from lymphocytes by using Dynabeads mRNA DIRECT Kit (Dynal, Oslo, Norway).

### RNA extraction and cDNA synthesis

We obtained tumor specimens of about 200 mg from breast carcinoma patients from discarded surgical samples, which were immediately frozen in liquid nitrogen.

Total RNA from frozen tumor specimens was prepared by using Total RNA Isolation System (Promega, Madison, WI) and subsequently used to purify mRNA using PolyATract mRNA Isolation System (Promega) according to manufacturer's instructions. About 500 ng of poly(A)^+ ^RNA from breast carcinomas or 1 μg of the poly(A)^+ ^RNA from peripheral blood lymphocytes were used to synthesize full-length cDNAs by using a SMART cDNA Library Construction Kit (Clontech, Palo Alto, CA).

### Analysis of antibody gene expression by PCR

The hypervariable V(D)J antibody region was amplified by PCR from cDNA templates by using site-specific primers 5'-GGA CAC GGC T(G/C) TGT ATT ACT G-3' and 5'-GCT GAG GAG ACG GTG ACC-3', designed in a study by Hansen and colleagues [27]. IgG1, IgG2 and IgA subclass determination was done as described earlier [39] by individually combining constant region-specific primers for IgG1, IgG2 and IgA genes (CG1d, CG2a and CA1, respectively) with a set of variable heavy chain primers: VH135, VH3a, VH3f, VH4, VH4b. These primers were designed for construction of human Fab libraries [40].

### ScFv library construction

The antibody gene repertoire was amplified using a set of primers designed for amplification of V_H _and V_L _antibody domains [41], and scFv fragments were assembled *in vitro *as described earlier [41]. The scFv fragments were then amplified by PCR with appropriate extension primers, incorporating *Nco*I, *Not*I restriction sites, facilitating the cloning of the scFv genes into the pKM19 vector. The resulting PCR products were purified by agarose gel electrophoresis (NuSieve 3:1 agarose, Rockland, ME). The DNA fragments were digested with *Nco*I/*Not*I and inserted into the pKM19 digested vector. Ligated DNA was used to transform competent DH5αF' bacterial cells (*supE44 *Δ *lac*U169 (*φ *80 *lacZ*ΔM15) *hsdR*17 *recA*1 *endA*1*gyrA*96 *thi*-1 *relA*1 F' [*traD*36 *proAB*^+ ^*lacI*^q^*lacZ*ΔM15]) by electroporation. The transformed cells were plated on 20 agar dishes (ø 15 cm), containing LB agar, 100 μg/mL ampicillin and 1% glucose. After overnight incubation at 37°C, bacterial colonies were scraped from the plates and resuspended in LB, containing 10% glycerol. Aliquots of this cell suspension were stored at -80°C and used for phage amplification.

### Phage amplification

Forty μL of scraped bacterial cells were incubated in 40 mL of LB containing ampicillin and 1% glucose until O.D. = 0.2. The bacteria were collected by centrifuging and resuspended in 40 mL of LB with ampicillin without glucose. About 6 × 10^9 ^PFU (plaque-forming units) of helper M13K07 were added to each mL of cell suspension, incubated for 15 min at 37°C without agitation and for another two h in a shaker. Kanamycin was added to obtain a final concentration of 20 μg/mL, and cells were incubated overnight at 32°C. Phage was purified according to standard PEG/NaCl precipitation [42].

### Cell-based selection of antibodies from phage-displayed library

MCF7 semiconfluent cells (about 2 × 10^7^) were rinsed three times with PBS and incubated with 2 mL of 2 mM EDTA in PBS for 15 min at 37°C. Ten mL of PBS containing 10 mM MgCl_2 _were added to the cells, which were accurately collected by pipetting. The cells were pelleted by centrifuging, washed once with 10 mL of PBS/MgCl_2 _and finally resuspended in 1 mL of freshly prepared blocking buffer: 4% non-fat dry milk, 0.05% Tween 20, 5 × 10^11 ^PFU of f1 UV-killed phage in PBS. The cells were blocked for 30 min at RT on rotating wheel, then collected and incubated for one h at 37°C on the wheel with about 5 × 10^11 ^TU (transducing units) of freshly amplified scFv antibody library in 1 mL of blocking buffer. The cells were washed five times with PBS/Tween and the bound phage eluted by adding 400 μL of 0.1 M HCl, pH 2.2 (adjusted by glycine). Cell suspension was incubated with elution solution for ten min at RT, neutralized by 40 μL of 2 M Tris-HCl, pH 9.6, and used for infection of bacterial cells. The bacteria were plated on two LB agar dishes (ø 15 cm), containing 100 μg/mL ampicillin and 1% glucose. Scraped bacteria were used for phage amplification.

### Affinity selection on purified protein targets

CEA and recombinant MUC1 were biotinylated as described earlier [43]. About 5 × 10^11 ^TU of freshly amplified scFv antibody libraries were preincubated with 50 μL of AD202 bacterial extract in blocking buffer for 30 min at 37°C. Twenty μg of a biotinylated protein (CEA, MUC1 or ED-B domain) were added to the reaction mixture and incubated for another h at 37°C under gentle agitation. The bound phage was captured by using streptavidin-coated Dynabeads M-280 (112.05, Dynal) according to manufacturer's instructions, washed five to ten times with PBS/Tween, then eluted and amplified as above.

### ELISA

The cells were grown in a 96-well plate until almost confluent. After discarding the growth medium, 100 μL of freshly prepared 4% paraformaldehyde (#15710, Electron Microscopy Science, Hatfield, PA) in PBS were rapidly added to well and incubated for ten min. The fixing solution was removed by pipetting and cells were incubated with blocking buffer (5% milk, in PBS) for 30 min at RT. PEG-purified phage in blocking buffer (1:1) was added to the cells and incubated for one h at 37°C under gentle agitation. The cells were washed three times with washing buffer (0.05% Tween 20 in PBS) and incubated with an anti-M13 HRP-conjugated antibody (27-9421-01, Amersham Biosciences, Piscataway, NJ) for 30 min at 37°C. The cells were washed five times and the immunoreaction developed by incubation with TMB liquid substrate (T8665, Sigma) for 15 min at RT and stopped by the addition of 25 μL 2 M H_2_SO_4_. The results were expressed as the difference between absorbances at 450 and 620 nm, determined by an automated ELISA reader. All assays were done in triplicate.

To test phage-antibody reactivity against soluble antigens, a protein solution at a concentration of 10 μg/mL in 50 mM NaHCO_3_, pH 9.6, were coated overnight at 4°C into Multiwell plates (Immunoplate Maxisorb, Nunc, Roskilde, Denmark). After discarding coating solution, plates were blocked for one h at 37°C with blocking buffer (5% milk, 0.05% Tween 20 in PBS). Plates were washed several times with washing buffer (0.05% Tween 20 in PBS). PEG-purified phage in blocking buffer (1:1) was added to each well and incubated for one h at 37°C. The immunoreaction was developed as above. All assays were done in duplicate.

### Soluble antibody production

Once identified, the *scFv *genes were recloned in pKM16 [35] for production of soluble antibodies. This plasmid directs protein expression under the control of the *lacP *promoter. The unique *Nco*I and *Not*I cloning sites facilitate insertion of antibody genes, allowing for expression of single-chain antibodies as fusion to the leader peptide and the first two amino acids of bacterial alkaline phosphatase, at the antibody's amino terminus, and as fusion to FLAG/6His-tail at the antibody's carboxy terminus.

A single colony was inoculated into 50 mL of LB containing 100 μg/mL Ap and 2% glucose. The culture was grown at 37°C for two to three h until O.D._600 _= 0.8. The cells were recovered by centrifugation, resuspended in 50 mL of fresh LB with 100 μg/mL Ap, 1 mM IPTG, 20 mM MgCl_2_, and incubated overnight at 30–32°C. Bacterial cells were pelleted and then resuspended in 0.5 mL of PBS. After three cycles of freeze and thaw, cell debris was pelleted and the soluble antibodies purified from the resulting supernatant by using HIS-Select HF Nickel Affinity Gel (H-0537, Sigma), according to manufacturer's instructions.

### Immunohistochemistry

To study the specificity of scFvs, about 10^5 ^cells were spun down onto each poly-L-lysine-covered glass slide. The slides were processed according to standard protocols and binding revealed using Vectastain ABC (Vector Laboratories, Burlingame, CA). Briefly, the cells were fixed for 20 min with 4% formaldehyde at RT or for ten min with cold methanol at -20°C, while endogenous peroxidase was blocked with 3% H_2_O_2 _in PBS for five min. After two washes with PBS, slides were blocked with 3% BSA in PBS for 30 min and then incubated for one h at RT (with 10 μg/mL of scFv). The slides were washed again and incubated for one h at RT with 10 μg/mL anti-FLAG HRP-conjugated monoclonal antibody (Sigma). After further washing, the slides were incubated with avidin-biotin-peroxidase complex for 30 min. Finally, DAB substrate (Vector Laboratories) was added and the reaction was stopped after two to ten min by washing in tap water. Counterstaining was performed with Mayer's hematoxylin (Vector Laboratories) for ten s. Then the slides were dehydrated by emerging into 75%, 80%, 95% and 100% ethanol solutions and clarified twice for two min in histolemon (Carlo Erba, Milan, Italy).

Before processing the non-fixed cells, the slides were air-dried for ten min and then used for staining.

Frozen tissue slices were thawed for one h at RT and hydrated with PBS. The slices were fixed with acetone for 10 min at RT and blocked with blocking solution (1.5% horse serum in PBS) for 30 min. The scFv antibodies, in a concentration of 10 μg/mL in blocking solution, were added to the slides for 30 min. The slides were then processed as above.

### Immunofluorescence staining

The cells were grown in a 24-well plate for cell culture (Nunc, Roskilde, Denmark), fixed with 3.7% formaldehyde in PBS for 10 min at RT and blocked with 3% BSA in PBS for one h at RT. PEG-purified phage in 1% BSA/PBS was added to the cells and incubated for one h under gentle agitation at 37°C. The cells were washed three times with 1% BSA in PBS and incubated with an anti-M13 mouse monoclonal antibody (27-9420-01, Amersham Biosciences) for 30 min at 37°C. The cells were washed as above and then incubated with an FITC-conjugated anti-mouse goat polyclonal antibody (554001, BD Biosciences Pharmingen, San Jose, CA) at a concentration of 5 μg/mL for 30 min at 37°C under gentle agitation. After the last incubation, cells were washed five times, dried in the dark, mounted with Vectashield medium (Vector Laboratories, Inc. Burlingame, CA) and cover glasses and analyzed using an inverted fluorescence microscope.

### Fluorescence-activated cell sorting (FACS) analysis

One hundred μL of phage suspension in TE (about 3 × 10^10 ^TU) were preincubated with 50 μL of 4% non-fat dried milk in PBS buffer for 15 min at RT under agitation to block unspecific binding. The phage sample was then added to 5 × 10^5 ^human cells in 50 μL of 1% BSA in PBS and incubated for one h at 4°C in a 96-well plate. After two washings with 1% BSA in PBS, a murine anti-M13 monoclonal antibody, diluted 1/50, was added to cell pellet and incubated for 30 min at 4°C. Afterwards, the cells were washed as above and incubated with an anti-mouse PE-conjugated antibody (550589, BD Biosciences Pharmingen), diluted 1/100, for another 30 min at RT. After staining, the cell samples were washed twice. Specific binding of the phage particles displaying scFv antibodies was measured by FACSArray or FACSCalibur instrument (BD Biosciences, Franklin Lakes, NJ). Viability detection was performed by adding 2.5 μL of 7-AAD staining solution (559925, BD Biosciences Pharmingen) to each sample.

For FACS analysis with soluble scFv antibodies about 5 × 10^5 ^cells were resuspended in 1% BSA in PBS and incubated, first with 2 μg of a purified scFv primary antibody, then with an anti-Flag antibody (F3165, Sigma) and finally with a FITC-labeled anti-mouse-IgG (F0257, Sigma), following manufacturer's instructions. Incubation was performed for 30–45 min in ice-water bath, the cells washed between steps with 1% BSA in PBS.

In case of intracellular staining, the cells were fixed by 4% cold formaldehyde in PBS for 5 min in ice and resuspended in 1% BSA, 0.5% Saponin (S1252, Sigma) in PBS. The cells were processed as above and washed between the steps with 1% BSA, 0.5% Saponin in PBS. Final washings were performed reducing the amount of saponin from 0.1 to 0.001 and then 0%. The samples resuspended in PBS were then acquired and analyzed by Cell quest software on FACSCalibur.

## Results

### Characterization of the lymphoplasmacytic cell infiltrates in breast tumor samples

We examined ten tumor specimens from breast cancer patients (aged 47–79) for presence and nature of TIL-B, first by PCR amplification of V(D)J antibody segments (CDR3, complementarity-determining region 3) and then by a comparison between IgG and IgA antibody classes presenting in tumor samples.

**Figure 1:**
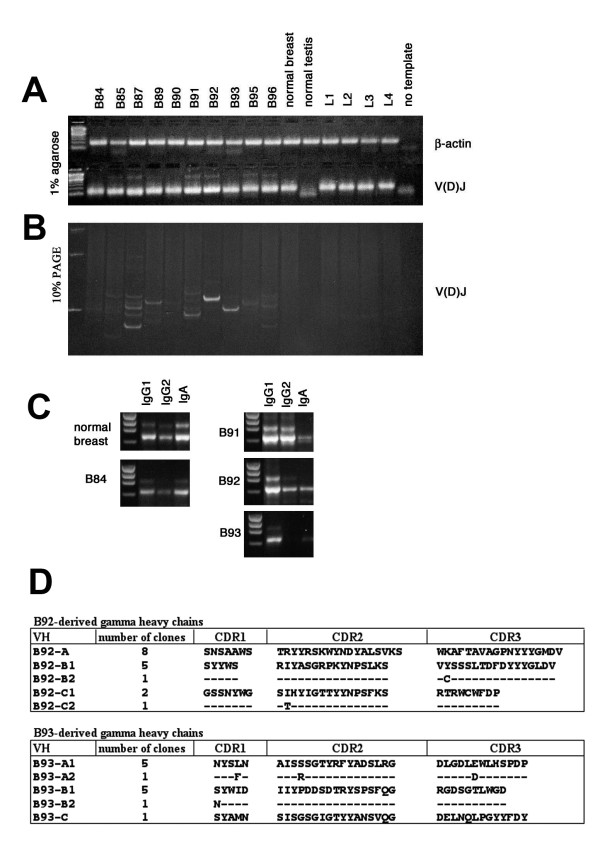
**Analysis of oligoclonality of TIL-derived antibodies**. ***(a) ***V(D)J analysis of TIL-derived antibody genes. SMART cDNA derived from ten different tumor samples (patients B84, B85, B87, B89, B90, B91, B92, B93, B95, B96), normal breast, normal testis and peripheral blood lymphocytes from four healthy donors (L1, L2, L3, L4), was used, as template for amplification of V(D)J antibody regions. Samples of cDNA were normalized by amplification of β-actin housekeeping gene. All V(D)J fragments were well-amplified and gave DNA bands of expected molecular weight in all cases, excluding normal testis cDNA sample. ***(b) ***The same PCR products were fractionated by 10% PAGE, giving a higher resolution of DNA bands. ***(c) ***Antibody subclass distribution. PCR-amplified normal breast and B84 cDNA samples not showing oligoclonal bands in V(D)J test, have prevalence of IgA bands in comparison to IgG1 and IgG2 (*left panels*), while three samples, B91, B92 and B93, giving strong oligoclonal bands in previous test, have IgG1 or both IgG1 and IgG2 band prevalence in comparison with IgA (*right panels*). ***(d) ***Clonality of heavy chain antibodies derived from B92 and B93 cDNA samples. Amino acid sequences of variable regions of 30 clones were deduced from randomly sequenced γ-chain antibody genes derived from B92 and B93 cDNA. Peptide sequences are reported in single-letter code. Identical amino acids in similar clones are represented by a dash.

We analyzed the expression patterns of the antibody fragment genes by semi-quantitative PCR from SMART cDNA template. A panel of cDNAs from ten breast carcinomas and samples of normal breast, testis and peripheral blood lymphocytes from healthy donors were normalized by PCR amplification of β-actin, a housekeeping gene (Fig. 1a). Hypervariable heavy chain antibody regions (V(D)J) were amplified as described in Materials and Methods. After analysis by agarose gel electrophoresis, the PCR samples were also fractionated by high resolving 10% PAGE (Fig. 1b). In applying this technique, we observed that seven of ten tumor-derived samples contained various discrete bands, characterizing oligoclonality of the immune response in these patients, while the well-amplified normal breast and peripheral lymphocyte DNA fragments did not contain intensive bands but formed a smear, consisting of bands of differing length. The observed oligoclonality of the immunoglobulins did not correlate with the age of the patients.

To analyze antibody subclass distribution we amplified Ig genes from breast carcinoma cDNA and normal breast, using subclass-specific primers. In agreement with the previous assay, the 3 cDNA tumor samples without oligoclonal bands in PCR-amplified V(D)J regions, had, in this test, a prevalence of IgA in comparison with IgG1 and IgG2 bands, just as in a sample of normal breast where IgA generally represents the major Ig class [44]. On the other hand, samples showing oligoclonality in the first assay contained IgG1, or both IgG1 and IgG2 as dominant antibody bands, in contrast to normal breast. Fig. 1c shows four more characteristic examples, along with normal breast sample. The cDNA samples from patients B85, B87, B91, B92, B93, B95 and B96 were chosen for library construction. Sample B85, which did not provide strong oligoclonal bands, nevertheless showed a prevalence of IgG antibodies (data not shown).

### Oligoclonality of TIL-B-derived antibodies in breast cancer patients was confirmed by sequencing

We chose two cDNA samples (B92, B93) that gave the strongest sharp bands in V(D)J test, for sequencing analysis. The nucleotide sequences of 17 and 13 randomly picked clones containing heavy chain genes deriving from B92 and B93 cDNA, respectively, were determined and their amino acid sequences deduced (Fig. 1d). All 30 clones encoded in-frame correctly organized heavy chains. The antibody clones, B92A and B93A, occurring more frequently, contained V(D)J regions of a length exactly corresponding to the strong bands observed earlier in Fig. 1b (lines with PCR products deriving from B92 and B93 samples) (data not shown), thus indicating that both PCR amplification with variable heavy chain primers and the cloning step do not introduce any particular bias interfering with heavy chain frequencies in generated libraries.

Six somatic mutations, identified in antibody fragments isolated many times, were localized within variable CDRs of V_H_s of the same specificity, while only one mutation was found in FRs of 30 heavy chain sequences (*P *= 0.0002). Therefore, the oligoclonality of the antibody repertoire, deriving from a tumor microenvironment, is a natural immune response occurring within tumor tissue driven by tumor antigens, and not an artifact introduced by PCR amplification.

### Library construction

Four scFv antibody libraries were generated using seven cDNA samples, characterized by oligoclonality of the immune response (see list of libraries in Table 1). Only the scFvEC23 library was constructed earlier [35] from peripheral blood lymphocytes, obtained from a single patient with advanced breast cancer.

**Table 1:** List of libraries and mixtures

**Library**	**Source of Ig genes**	**Patient(age)**	**Library complexity**
scFvB87	TIL	B87 (55)	4.7 × 10^5^
scFvB95	TIL	B95 (73)	1.1 × 10^7^
scFvB96	TIL	B96 (72)	2.6 × 10^7^
scFvmix	TIL	B85 (47), B91 (70), B92 (79), B93 (66)	2.4 × 10^7^
scFvEC23	PBL	EC23 (65)	1.8 × 10^7^
mixTIL: scFvB87, scFvB95, scFvB96, scFvmix	TIL		
mixLIB: scFvB87, scFvB95, scFvmix, scFvEC23	TIL+PBL		

We used a novel pKM19 vector for construction of the libraries [35]. This vector is characterized by the following features: (i) use of the PhoA leader peptide (a genuine *E. coli *periplasmic protein) guarantees efficient membrane assembly and processing of recombinant antibodies; (ii) relatively low antibody expression levels prevent abundant protein production, reducing biological bias for harmful antibodies that may affect bacterial growth or be toxic for host bacteria, thus increasing the actual complexity of a generated library; (iii) fusion of scFv antibody to deleted gpIII protein improves antibody display efficiency in this system.

### Selection of specific antitumor antibodies from phage display libraries generated from TIL-B and PBL

**Figure 2:**
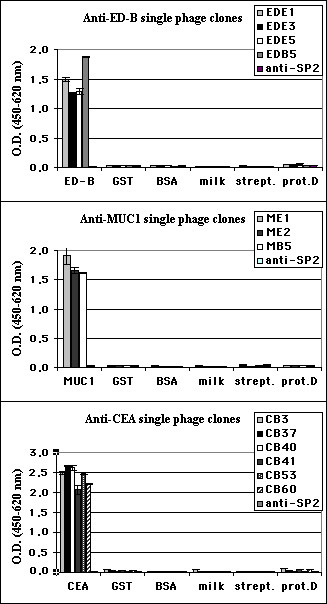
**ELISA reactivity of single phage antibodies**. Reactivity of single phage clones after third round of selection against ED-B, MUC1 and CEA was tested using respective proteins. Data reported are the average values of assays performed in duplicate. Several irrelevant proteins and an anti-SP2 irrelevant phage antibody [35] are included as negative controls.

We examined the possibility of selecting specific antibody fragments from phage libraries against common cancer antigens available in our lab, including ED-B domain of fibronectin, MUC1 epithelial mucin, and CEA. Under conditions described in Materials and Methods, a mixture of four TIL-derived scFv libraries (mixTIL) and the scFvEC23 library were panned separately against three protein targets in several rounds. In every case, we observed that phage pools were positive against the selecting antigen already after the second and third panning rounds (data not shown). Randomly picked clones from pools of phage after the third round of selection were tested in ELISA for binding reactivity against the respective antigens. Positive clones were analyzed by DNA fingerprinting using *Hae*III and *Alu*I double digestion and all the various antibody clones were sequenced. Table 2 summarizes the clone analysis data. Fig. 2 represents ELISA of single scFv phages selected on purified antigens.

**Table 2:** Results of selection by using purified tumor antigens

**Target antigen**	**Library**	**ELISA positive clones/tested clones**	**Isolated antibody genes**
ED-B	mixTIL	10/10	1
ED-B	scFvEC23	10/10	3
MUC1	mixTIL	2/16	1
MUC1	scFvEC23	6/8	2
CEA	mixTIL	13/16	6

### Cell-based selection of tumor-specific antibodies

**Figure 3:**
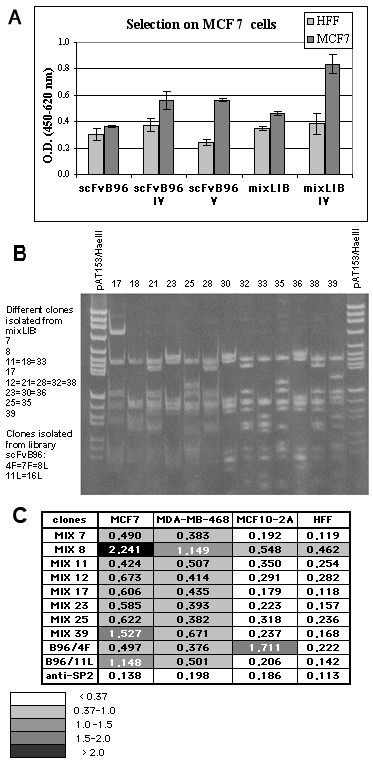
**Selection of anti-MCF7 antibodies**. ***(a) ***Reactivity of phage pools after fourth and fifth rounds of panning, in comparison with original libraries, was tested. Data reported are the average values of assays performed in triplicate. ***(b) ***Fingerprinting analysis of antibody clones. PCR-amplified scFv genes were analyzed by using *Hae*III and *Alu*I double digestion. The analysis of clones 17–39, selected from mixLIB, is shown at right, and the list of different anti-MCF7 antibodies obtained shown at left. ***(c) ***Cell ELISA reactivity of single phage clones. Data reported are the average values of assays performed in triplicate. Cells developing with irrelevant anti-SP2 antibody are included as negative control.

We tested functionality of a single TIL-derived library (scFvB96) by selecting breast cancer-specific antibodies through cell-based panning on living MCF7 breast carcinoma cells. Four additional libraries, including scFvB87, scFvB95, scFvmix and scFvEC23, were pooled together (mixLIB) and panned in similar fashion. Four or five selection rounds on the tumor cells were necessary for mixLIB and scFvB96 libraries, respectively, in order to obtain phage pools enriched by specific cell binders (Fig. 3a). Then, randomly picked clones were analyzed by PCR for presence of complete scFv antibody genes. The full-length scFv phage clones were tested by cell-based ELISA, and analyzed by DNA fingerprinting (Fig. 3b). Table 3 summarizes clone analysis data. All different positive clones were sequenced. Amino acid sequences, deduced from DNA sequences, confirmed correct, in-frame antibody structures.

**Table 3:** Results of cell-based selection

	**MCF7-based selection**	
Library	scFvB96	mixLIB
Round of selection	5	4
Full-length scFvs/PCR-tested clones	12/40	30/40
ELISA positive/full-length clones	5/12	22/30
Isolated antibody genes	2	8

The reactivity and specificity of cell-selected antibodies were verified by ELISA on two breast carcinoma cell lines: MCF7, MDA-MB-468 and normal cells, as negative controls: MCF10-2A (normal human breast epithelium), HFF (human foreskin fibroblasts) (Fig. 3c). Of ten different antibodies belonging to seven specificity groups (MIX7, MIX12, MIX25 have the same heavy chain sequence and different light chains; MIX8 and MIX39 have similar sequences with minor differences), nine scFvs specifically bind to breast carcinoma cells, while the B96/4F antibody alone also binds to normal epithelial cells as well.

### Cell-selected antibodies derived from TIL

**Figure 4:**
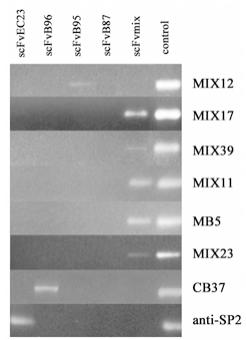
**Origin of anti-MCF7 scFv antibodies**. One μL of each scFv phage library was amplified by PCR using oligonucleotide primers specific for analyzed antibody genes. Corresponding PEG-purified phage was used as positive control (*last line*). The irrelevant anti-SP2 antibody gene of known origin, selected earlier from scFvEC23 library, was also tested.

MIX7-MIX39 scFv antibodies were selected from a mixture of PBL and TIL-derived libraries. We investigated the origin of these antibodies to see which type of library functions better under equal selection conditions. One μL of each amplified library was used as template for PCR amplification with a pair of oligonucleotide primers specific for each antibody (Fig. 4). This analysis shows that five tested scFv antibodies, isolated from a mixture of libraries, belong to TIL-derived antibodies. Antibody genes of MIX7 and MIX25 (having the same heavy chain as MIX12), and MIX8 (similar to MIX39) are believed to have a similar origin. Regarding the irrelevant anti-SP2 antibody, selected earlier from an scFvEC23 library [35], its origin from a PBL-derived library was confirmed.

### Staining of tumor cells

**Figure 5:**
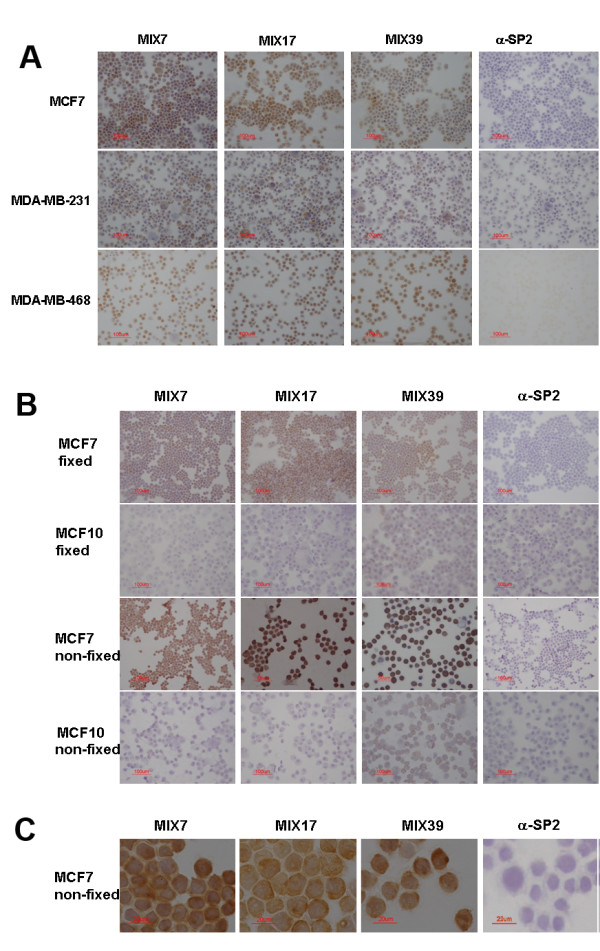
**Immunohistochemical staining of tumor cells**. ***(a) ***The scFvs MIX7, MIX17 and MIX39 show significant staining of the MCF7, MDA-MB-231 and MDA-MB-468 breast carcinoma cells. No staining is observed with the negative control (irrelevant anti-SP2 antibody). ***(b) ***Staining of breast carcinoma cells in comparison with normal breast epithelial cells MCF10-2A. The selected antibodies stain the non-fixed cells more intensively than the fixed MCF7 cells, but not the MCF10-2A cells. Weak background is observed only for MIX39 scFv when it interacts with MCF10-2A cells. No staining is observed for negative control anti-SP2 antibody. ***(c) ***Staining non-fixed MCF7 cells, magnification ×60.

To demonstrate the specificity of selected antibodies we examined three scFvs in soluble form (MIX7, MIX17 and MIX39) by immunohistochemical staining. These antibodies were chosen because of their good reactivity, specificity and stability in soluble form. The first staining was performed on various methanol-fixed breast carcinoma cells, including MCF7, MDA-MB-231 and MDA-MB-468 (Fig. 5a). Different intensity staining was observed for all three antibodies tested, compared to the irrelevant anti-SP2 antibody. In the second experiment, formaldehyde-fixed or non-fixed dried cells were stained (Fig. 5b). All selected antibodies specifically stained both fixed and non-fixed carcinoma MCF7 cells, but did not stain normal epithelial MCF10-2A cells. However, the signal was notably stronger for non-fixed cells. Weak background labeling was registered for MIX39 when it interacted with non-fixed MCF10-2A cells. Intensive staining activity of MIX17 and MIX7 was associated with the cell membrane, cytoplasm and nuclear membrane, while MIX39 staining was of nuclear localization (Fig. 5c).

**Figure 6:**
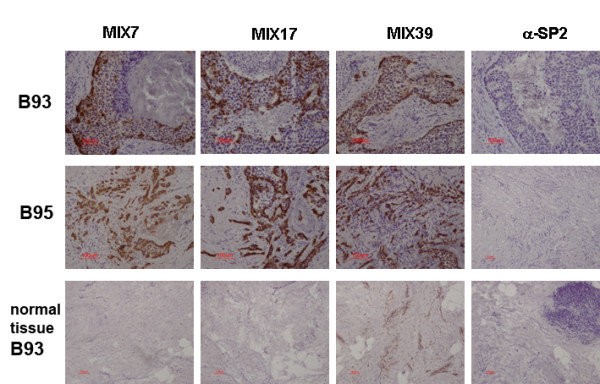
**Immunohistochemical staining of tumor tissues**. Slices of breast tumor tissue from patient B95 and matched breast tumor and normal tissues from patient B93 were stained with MIX7, MIX17 and MIX39 soluble antibodies and an irrelevant anti-SP2 antibody. Intensive staining tumor tissues were observed for all selected antibodies. MIX39 slightly stains matched normal breast tissue of patient B93.

We also stained tumor and normal matched breast tissues, available in our laboratory, from patients B93 and B95. All the scFvs tested strongly stained tumor cells and were negative with normal matched tissue from patient B93 (Fig. 6). The irrelevant anti-SP2 antibody did not react with the tissue slices tested.

**Figure 7:**
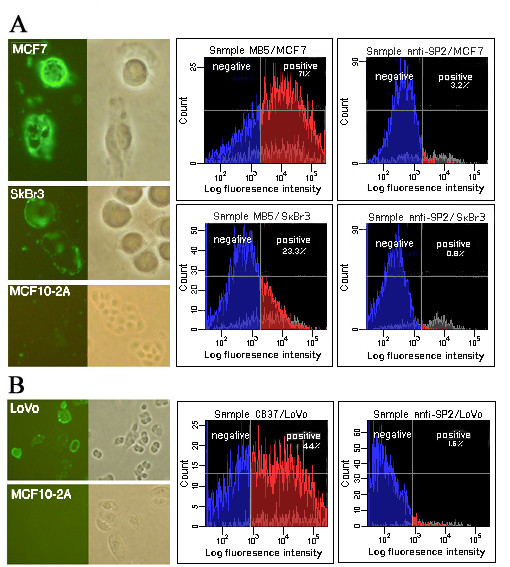
**Fluorescence staining and flow cytometry with anti-MUC1 and anti-CEA antibodies**. ***(a) ***Fluorescence staining of breast carcinoma cells MCF7 and SkBr3, expressing epithelial mucin MUC1 and normal breast epithelial MCF10-2A cells by using phage antibody anti-MUC1 MB5 (*left panels*). *Right panels *show results of flow cytometry analysis of phage displayed MB5 and irrelevant anti-SP2 single-chain antibodies. ***(b) ***Staining of LoVo colorectal adenocarcinoma cells expressing CEA protein by phage-displayed anti-CEA CB37 scFv antibody is shown. Staining of negative control MCF10-2A cells is included (*left panels*). Binding of phage antibody CB37 to LoVo was also measured by flow cytometry (*right panels*).

The binding capacity of the anti-MUC1 antibody MB5 and the anti-CEA antibody CB37 were assessed by immunofluorescence staining of tumor cells directly with phage antibodies (Fig. 7). The MB5 antibody intensively stained MCF7 cells, known for high MUC1 expression, and also reacted well with another breast carcinoma cell line, SkBr3. The CB37, an anti-CEA antibody, efficiently bound colon adenocarcinoma cells, LoVo, expressing the carcinoembryonic antigen. No background staining for normal breast epithelium was observed. Binding of the MB5 and CB37 phage-displayed scFvs was also measured by flow cytometry. According to FACS analysis, the MB5 stained 71% of MCF7 and 23.3% of ScBr3 cells. With regard to the anti-CEA CB37 antibody, it bound 44% of LoVo cells.

**Figure 8:**
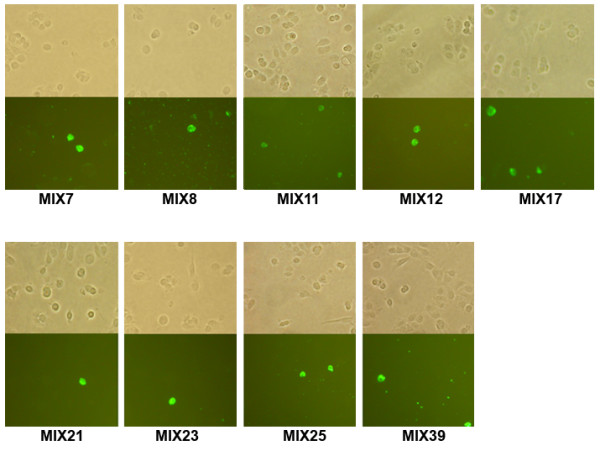
**Fluorescence staining with anti-MCF7 antibodies**. Fluorescence staining of breast carcinoma MCF7 fixed non-permeabilized cells by phage antibodies. No staining of negative control MCF10-2A cells was observed (data not shown).

All anti-MCF7 phage clones were tested in fluorescence staining of non-permeabilized MCF7 breast carcinoma cells (Fig. 8) in comparison with normal MCF10-2A cells (not shown). All antibodies stained only a low percentage of MCF7 cells, probably apoptotic or dead cells. No background staining for normal breast epithelium was observed. FACS analysis performed with three soluble scFv antibodies confirmed that MIX antibodies react with intracellular antigens of tumoral cells (Table 4).

**Table 4:** Flow cytometry analysis of two MCF7 and MDA-MB-468 tumor cell lines and normal breast epithelial MCF10-2A cells. Irrelevant antibody anti-SP2 was used as negative control, while an α-tubulin monoclonal antibody was used as positive control for intracellular staining

**Cell line/scFv antibody**	**Surface staining of alive cells**			**Intracellular staining**		
**MCF7**	% pos	MFI*	iMFI**	% pos	MFI	iMFI

α-tubulin	n.t.	n.t.	n.t.	88.04	684.49	60262.5
anti-SP2	2.73	24.45	66.7	0.36	4221.17	1519.6
MIX7	2.84	29.19	82.9	73.03	603.58	44079.4
MIX17	2.68	22.76	59.9	84.2	733.63	61771.6
MIX39	3.98	22.9	91.1	13.89	504.85	7012.4

**MDA-MB-468**	% pos	MFI	iMFI	% pos	MFI	iMFI

α-tubulin	n.t.	n.t.	n.t.	85.22	259.18	22087.3
anti-SP2	1.9	51.17	97.2	0.98	579.13	567.5
MIX7	3.93	40.4	158.8	37.14	214.39	7962.4
MIX17	5.38	33.04	177.8	70.54	260.36	18365.8
MIX39	3.33	30.04	100.0	2.51	237.64	596.5

**MCF10-2A**	% pos	MFI	iMFI	% pos	MFI	iMFI

α-tubulin	n.t.	n.t.	n.t.	42.05	169.64	7133.4
anti-SP2	0.44	95.68	42.1	0.44	1515.5	666.8
MIX7	0.94	208.51	196.0	0.29	962.79	279.2
MIX17	0.81	11.84	9.6	1.06	824.83	874.3
MIX39	0.93	175.42	163.1	6.49	156.57	1016.1

* mean fluorescence intensity

** integrated MFI (% pos × MFI)

n.t.- not tested

## Discussion

In comparison with the nondetectable clonality of B cells in peripheral blood lymphocytes (<1/20,000), B cells from tumor-draining lymph nodes and tumor-infiltrating lymphocytes represent a much more limited Ig repertoire [45]. About 7% of lymph node-derived, and between 18–68% of TIL-derived, heavy chain antibody sequences belong to clonal groups [45], thus indicating both tumor-draining lymph nodes and tumor-infiltrating lymphocytes as promising sources of tumor-specific antibodies. In fact, identification of anti-tumor recombinant antibodies from display libraries derived from lymph nodes of cancer patients was reported in several studies, as mentioned in the Introduction [14,17,18]. However, we found it quite difficult to obtain, as fresh surgical material, metastasized or tumor-draining lymph nodes from breast cancer patients. According to recent medical practice, the surgeon removes only a sentinel lymph node, or a small cluster of nodes (sentinel node and those closest to it), instead of removing dozens of lymph nodes as before, thus performing less invasive surgery and reducing side effects. After sentinel lymph node dissection, practically the entire node is studied for presence of micrometastasis or single cancer cells. As a result, in breast cancer surgery, the dissected node is virtually unavailable as discarded surgical material.

In this article, we examined the possibility of using primary tumors as a source of genes of antitumor antibodies, potentially useful for diagnostic and therapeutic approaches. We showed, by PCR amplification of specific antibody gene regions deriving from ten primary breast tumors (none being of the rare MBC histological type) of patients aged between 49–79 years, that seven of ten of these samples (70%), have a prominent IgG antibody expression, as compared with the IgA subclass. This correlates with the oligoclonality of the hypervariable region of heavy chain antibodies, suggesting a specific immune response to tumor-expressed antigens. Clonality of tumor-derived antibodies was confirmed by sequencing analysis. The great majority of the gamma heavy chains, derived from TIL-B of B92 and B93 patients, belong to clonal groups. The higher frequency of somatic mutations observed within CDRs vs FRs in variable regions of heavy chains of the same specificity indicates that tumor-infiltrated B-cells locally produce a restricted IgG repertoire, with evidence of antigen-driven maturation.

We identified a panel of tumor-specific antibodies from the described libraries; these antibodies were reactive with ED-B domain, MUC1, CEA and MCF7 breast carcinoma cells used in the respective selections. It is interesting to note that, in cell-based selection, performed without a subtractive panning step on normal breast epithelium, and in contrast with numerous previously described selection protocols [17,18,46-48], we isolated only one nonspecific scFv, which recognized normal breast epithelium as well. This probably indicates that our modest-sized TIL-derived libraries, despite a very restricted antibody repertoire, contain quite strong easily selectable antitumor binders. The antibodies obtained in the cell-based selection, recognized intracellular antigens, as shown by fluorescent and immunohistochemical staining, and flow cytometry analysis. This result agrees with Hoogenboom's findings, which demonstrated that local humoral immune response in colorectal carcinoma patients was biased toward intracellular target antigens [29]. Notably, that antibody selection from a mixture of PBL- and TIL-derived libraries clearly shows the latter to be more efficient in cell-based panning. In fact, all isolated anti-MCF7 single-chain antibodies appeared to be derived from tumor-infiltrating lymphocytes. The libraries derived from TIL have quite low complexity, as it was shown by random sequencing of antibody repertoire in two patients (Fig 1d). For this reason, we presume that the efficient selection of scFvs against various antigens tested is a result of the strong antitumor profiles of such libraries and the use of a suitable combination of antibody repertoires from various patients. In order to understand how often antibodies against a single antigen occur in different TIL-derived repertoires, we attempted to select new anti-CEA antibodies from a new mixture of libraries, this time excluding the scFvB96 library, since one of the anti-CEA antibodies, CB37, derived exactly from this library (Fig. 3c). No new antibodies were selected, indicating that patient B96 alone, among seven patients, had local immune response against the CEA antigen. However, in order to reach a definitive conclusion on the capacity of TIL-derived libraries, it would be interesting to compare selection against the same antigens from TIL- and PBL-derived libraries from the same patient. Unfortunately, we have no such matched libraries.

To sum up, TIL-derived libraries gave good results in all performed selections, providing a panel of human tumor-specific antibodies which recognize tumor cell surface and intracellular antigens.

As mentioned in the Background, the selection of specific antitumor antibodies from TIL-derived phage-display libraries often failed, while an alternative approach, based on a phage-expression tumor-derived library and direct plaque screening protocols that avoided the limitations of a phage display system, allowed Wu and colleagues [49] to isolate multiple antibodies that specifically bind cultured tumor cells. In the present study, we applied a novel pKM19 vector for display of recombinant antibodies in single-chain format. We believe the application of the improved display system permitted us to generate the functional tumor-derived phage-display libraries, giving rise to various antibodies that recognize tumor cell antigens.

## Conclusion

Our results indicate that natural immune responses to tumor-related antigens occur quite frequently in patients with breast cancer, not only in histologically-defined MCB. Tumor samples as small as 0.2 g, obtained as surgical material, can be exploited as an appropriate source for generation of recombinant phage display libraries enriched for tumor-specific antibodies. Isolation of a panel of antitumor scFvs through selection against desirable protein targets, as well as against living breast carcinoma cells, shows this approach to be very promising for development of human antibodies, potentially useful for diagnostic and therapeutic approaches.

Moreover, investigation of the protein targets eliciting production of tumor cell-specific antibodies in a tumor microenvironment may (i) provide important information about individual immunoreactivity of a given patient, affording a prognostic value; (ii) open an ample perspective for discovery of novel tumor-specific antigens.

## List of abbreviations used

BSA – bovine serum albumin; CDRs – complementarity-determining regions; ELISA – enzyme-linked immunosorbent assay; FACS – Fluorescence-activated cell sorting; HRP- horseradish peroxidase; Ig – immunoglobulin(s); MCB – medullary carcinoma of breast; PBL – peripheral blood lymphocytes; PBS – phosphate-buffered saline; PEG – polyethylene glycol; PFU – plaque-forming unit(s); RT – room temperature; TIL-B – tumor-infiltrating B lymphocytes; TU – transducing unit(s)

## Competing interests

All author(s) receive salaries from Sigma-Tau, SpA, the organization which holds the patent relating to the content of this manuscript.

## Authors' contributions

EP, GM and DS contributed to library construction, recombinant antibody selection and fluorescent staining experiments. AMA, AP and VDA contributed to immunological analysis of selected recombinant antibodies. FP and RDS planned and coordinated characterization of antitumor antibodies. OM, who coordinated the entire project and prepared the manuscript, performed gene expression analysis and promoted the TIL-B-based approach. All authors read and approved the final manuscript.

## References

[B1] Kohler G, Milstein C (1975). Continuous cultures of fused cells secreting antibody of predefined specificity. Nature.

[B2] Milenic DE (2002). Monoclonal antibody-based therapy strategies: providing options for the cancer patient. Curr Pharm Des.

[B3] Ross JS, Gray K, Gray GS, Worland PJ, Rolfe M (2003). Anticancer antibodies. Am J Clin Pathol.

[B4] Maher VE, Drukman SJ, Kinders RJ, Hunter RE, Jennings J, Brigham C, Stevens S, Griffin TW (1992). Human antibody response to the intravenous and intraperitoneal administration of the F(ab')2 fragment of the OC125 murine monoclonal antibody. J Immunother.

[B5] McCafferty J, Griffiths AD, Winter G, Chiswell DJ (1990). Phage antibodies: filamentous phage displaying antibody variable domains. Nature.

[B6] Barbas CF, Kang AS, Lerner RA, Benkovic SJ (1991). Assembly of combinatorial antibody libraries on phage surfaces: the gene III site. Proc Natl Acad Sci USA.

[B7] Sheets MD, Amersdorfer P, Finnern R, Sargent P, Lindquist E, Schier R, Hemingsen G, Wong C, Gerhart JC, Marks JD (1998). Efficient construction of a large nonimmune phage antibody library: the production of high-affinity human single-chain antibodies to protein antigens. Proc Natl Acad Sci USA.

[B8] de Haard HJ, van Neer N, Reurs A, Hufton SE, Roovers RC, Henderikx P, de Bruine AP, Arends JW, Hoogenboom HR (1999). A large non-immunized human Fab fragment phage library that permits rapid isolation and kinetic analysis of high affinity antibodies. J Biol Chem.

[B9] Thompson J, Pope T, Tung JS, Chan C, Hollis G, Mark G, Johnson KS (1996). Affinity maturation of a high-affinity human monoclonal antibody against the third hypervariable loop of human immunodeficiency virus: use of phage display to improve affinity and broaden strain reactivity. J Mol Biol.

[B10] Pini A, Viti F, Santucci A, Carnemolla B, Zardi L, Neri P, Neri D (1998). Design and use of a phage display library. Human antibodies with subnanomolar affinity against a marker of angiogenesis eluted from a two-dimensional gel. J Biol Chem.

[B11] Marks JD, Hoogenboom HR, Bonnert TP, McCafferty J, Griffiths AD, Winter G (1991). By-passing immunization. Human antibodies from V-gene libraries displayed on phage. J Mol Biol.

[B12] Vaughan TJ, Williams AJ, Pritchard K, Osbourn JK, Pope AR, Earnshaw JC, McCafferty J, Hodits RA, Wilton J, Johnson KS (1996). Human antibodies with sub-nanomolar affinities isolated from a large non-immunized phage display library. Nat Biotechnol.

[B13] Lang IM, Barbas CF, Schleef RR (1996). Recombinant rabbit Fab with binding activity to type-1 plasminogen activator inhibitor derived from a phage-display library against human alpha-granules. Gene.

[B14] Clark MA, Hawkins NJ, Papaioannou A, Fiddes RJ, Ward RL (1997). Isolation of human anti-c-erbB-2 Fabs from a lymph node-derived phage display library. Clin Exp Immunol.

[B15] Yip YL, Hawkins NJ, Clark MA, Ward RL (1997). Evaluation of different lymphoid tissue sources for the construction of human immunoglobulin gene libraries. Immunotechnology.

[B16] Graus YF, Verschuuren JJ, Degenhardt A, van Breda Vriesman PJ, De Baets MH, Posner JB, Burton DR, Dalmau J (1998). Selection of recombinant anti-HuD Fab fragments from a phage display antibody library of a lung cancer patient with paraneoplastic encephalomyelitis. J Neuroimmunol.

[B17] Rothe A, Klimka A, Tur MK, Pfitzner T, Huhn M, Sasse S, Mallmann P, Engert A, Barth S (2004). Construction of phage display libraries from reactive lymph nodes of breast carcinoma patients and selection for specifically binding human single chain Fv on cell lines. Int J Mol Med.

[B18] Xu MY, Xu XH, Chen GZ, Deng XL, Li J, Yu XJ, Chen MZ (2004). Production of a human single-chain variable fragment antibody against esophageal carcinoma. World J Gastroenterol.

[B19] Sikora K, Alderson T, Phillips J, Watson JV (1982). Human hybridomas from malignant gliomas. Lancet.

[B20] Sikora K, Alderson T, Ellis J, Phillips J, Watson J (1983). Human hybridomas from patients with malignant disease. Br J Cancer.

[B21] Punt CJ, Barbuto JA, Zhang H, Grimes WJ, Hatch KD, Hersh EM (1994). Anti-tumor antibody produced by human tumor-infiltrating and peripheral blood B lymphocytes. Cancer Immunol Immunother.

[B22] Zhang H, Lake DF, Barbuto JA, Bernstein RM, Grimes WJ, Hersh EM (1995). A human monoclonal antimelanoma single-chain Fv antibody derived from tumor-infiltrating lymphocytes. Cancer Res.

[B23] Imahayashi S, Ichiyoshi Y, Yoshino I, Eifuku R, Takenoyama M, Yasumoto K (2000). Tumor-infiltrating B-cell-derived IgG recognizes tumor components in human lung cancer. Cancer Invest.

[B24] Yasuda M, Takenoyama M, Obata Y, Sugaya M, So T, Hanagiri T, Sugio K, Yasumoto K (2002). Tumor-infiltrating B lymphocytes as a potential source of identifying tumor antigen in human lung cancer. Cancer Res.

[B25] Kotlan B, Simsa P, Gruel N, Foldi J, Fridman WH, Petranyi G, Teillaud JL (2000). A scFv phage display mini library generated from the immunoglobulin repertoire of breast medullary carcinoma infiltrating B lymphocytes. Dis Markers.

[B26] Coronella JA, Telleman P, Kingsbury GA, Truong TD, Hays S, Junghans RP (2001). Evidence for an antigen-driven humoral immune response in medullary ductal breast cancer. Cancer Res.

[B27] Hansen MH, Nielsen H, Ditzel HJ (2001). The tumor-infiltrating B cell response in medullary breast cancer is oligoclonal and directed against the autoantigen actin exposed on the surface of apoptotic cancer cells. Proc Natl Acad Sci USA.

[B28] O'Brien PM, Tsirimonaki E, Coomber DW, Millan DW, Davis JA, Campo MS (2001). Immunoglobulin genes expressed by B-lymphocytes infiltrating cervical carcinomas show evidence of antigen-driven selection. Cancer Immunol Immunother.

[B29] Roovers RC, van der Linden E, Zijlema H, de Bruine A, Arends JW, Hoogenboom HR (2001). Evidence for a bias toward intracellular antigens in the local humoral anti-tumor immune response of a colorectal cancer patient revealed by phage display. Int J Cancer.

[B30] Coronella JA, Spier C, Welch M, Trevor KT, Stopeck AT, Villar H, Hersh EM (2002). Antigen-driven oligoclonal expansion of tumor-infiltrating B cells in infiltrating ductal carcinoma of the breast. J Immunol.

[B31] Kotlan B, Simsa P, Teillaud JL, Fridman WH, Toth J, McKnight M, Glassy MC (2005). Novel ganglioside antigen identified by B cells in human medullary breast carcinomas: the proof of principle concerning the tumor-infiltrating B lymphocytes. J Immunol.

[B32] Hammarstrom S (1999). The carcinoembryonic antigen (CEA) family: structures, suggested functions and expression in normal and malignant tissues. Semin Cancer Biol.

[B33] McGuckin MA, Walsh MD, Hohn BG, Ward BG, Wright RG (1995). Prognostic significance of MUC1 epithelial mucin expression in breast cancer. Hum Pathol.

[B34] Zardi L, Carnemolla B, Siri A, Petersen TE, Paolella G, Sebastio G, Baralle FE (1987). Transformed human cells produce a new fibronectin isoform by preferential alternative splicing of a previously unobserved exon. EMBO J.

[B35] Pavoni E, Monteriu G, Cianfriglia M, Minenkova O (2007). New display vector reduces biological bias for expression of antibodies in E. coli. Gene.

[B36] Soule HD, Maloney TM, Wolman SR, Peterson WD, Brenz R, McGrath CM, Russo J, Pauley RJ, Jones RF, Brooks SC (1990). Isolation and characterization of a spontaneously immortalized human breast epithelial cell line, MCF-10. Cancer Res.

[B37] Minenkova O, Pucci A, Pavoni E, De Tomassi A, Fortugno P, Gargano N, Cianfriglia M, Barca S, De Placido S, Martignetti A, Felici F, Cortese R, Monaci P (2003). Identification of tumor-associated antigens by screening phage-displayed human cDNA libraries with sera from tumor patients. Int J Cancer.

[B38] Smith DB, Johnson KS (1988). Single step purification of polypeptides expressed in *Escherechia coli *as fusion with glutathione S-transferase. Gene.

[B39] Hansen MH, Nielsen HV, Ditzel HJ (2002). Translocation of an intracellular antigen to the surface of medullary breast cancer cells early in apoptosis allows for an antigen-driven antibody response elicited by tumor-infiltrating B cells. J Immunol.

[B40] Barbas CF, Burton DR (1994). Monoclonal antibodies from combinatorial libraries. Cold Spring Harbor Laboratory Course Manual.

[B41] Pope AR, Embleton M, Mernaugh R, McCafferty J, Hoogenboom H, Chiswell D (1996). Construction and use of antibody gene repertoires. Antibody Engineering – A practical approach.

[B42] Sambrook J, Fritsch EF, Maniatis T (1989). Molecular cloning: A laboratory manual.

[B43] Harlow E, Lane D (1988). Antibody: A laboratory manual.

[B44] Drife JO, McClelland DB, Pryde A, Roberts MM, Smith II (1976). Immunoglobulin synthesis in the "resting" breast. Br Med J.

[B45] Coronella-Wood JA, Hersh EM (2003). Naturally occurring B-cell responses to breast cancer. Cancer Immunol Immunother.

[B46] Topping KP, Hough VC, Monson JR, Greenman J (2000). Isolation of human colorectal tumour reactive antibodies using phage display technology. Int J Oncol.

[B47] Ridgway JB, Ng E, Kern JA, Lee J, Brush J, Goddard A, Carter P (1999). Identification of a human anti-CD55 single-chain Fv by subtractive panning of a phage library using tumor and nontumor cell lines. Cancer Res.

[B48] Shadidi M, Sioud M (2001). An anti-leukemic single chain Fv antibody selected from a synthetic human phage antibody library. Biochem Biophys Res Commun.

[B49] Wu H, Pancook JD, Beuerlein G, Chilton T, Pecht G, Huse WD, Watkins JD (2002). Cloning, isolation and characterization of human tumor *in situ *monoclonal antibodies. Cancer Immunol Immunother.

